# AlphaKnot 2.0: a web server for the visualization of proteins’ knotting and a database of knotted AlphaFold-predicted models

**DOI:** 10.1093/nar/gkae443

**Published:** 2024-06-06

**Authors:** Pawel Rubach, Maciej Sikora, Aleksandra I Jarmolinska, Agata P Perlinska, Joanna I Sulkowska

**Affiliations:** Warsaw School of Economics, Al. Niepodleglosci 162, 02-554 Warsaw, Poland; Centre of New Technologies, University of Warsaw, Banacha 2c, 02-097 Warsaw, Poland; Centre of New Technologies, University of Warsaw, Banacha 2c, 02-097 Warsaw, Poland; Centre of New Technologies, University of Warsaw, Banacha 2c, 02-097 Warsaw, Poland; Centre of New Technologies, University of Warsaw, Banacha 2c, 02-097 Warsaw, Poland

## Abstract

The availability of 3D protein models is rapidly increasing with the development of structure prediction algorithms. With the expanding availability of data, new ways of analysis, especially topological analysis, of those predictions are becoming necessary. Here, we present the updated version of the AlphaKnot service that provides a straightforward way of analyzing structure topology. It was designed specifically to determine knot types of the predicted structure models, however, it can be used for all structures, including the ones solved experimentally. AlphaKnot 2.0 provides the user’s ability to obtain the knowledge necessary to assess the topological correctness of the model. Both probabilistic and deterministic knot detection methods are available, together with various visualizations (including a trajectory of simplification steps to highlight the topological complexities). Moreover, the web server provides a list of proteins similar to the queried model within AlphaKnot’s database and returns their knot types for direct comparison. We pre-calculated the topology of high-quality models from the AlphaFold Database (4th version) and there are now more than 680.000 knotted models available in the AlphaKnot database. AlphaKnot 2.0 is available at https://alphaknot.cent.uw.edu.pl/.

## Graphical Abstract

**Graphical Abstract:**
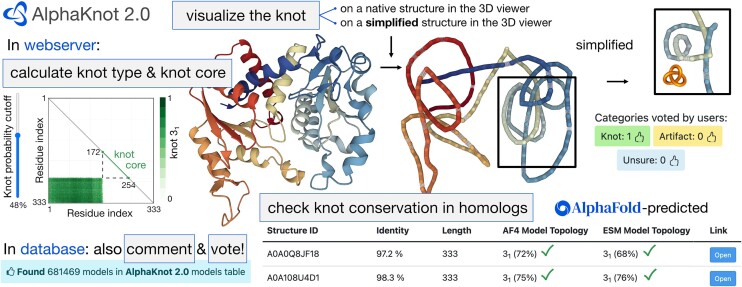

## Introduction

Machine learning models, especially large-scale ones, such as AlphaFold (1), ESMFold (2) or RoseTTAFold (3), make predicting 3D structures of proteins an enticing alternative to solving them experimentally. One can use them to predict the structure of a protein of choice, or just dive right into the wealth of structures already predicted by the creators to showcase the models’ capabilities (with AlphaFold Database (4) boasting more than 200 million modeled structures).

Such a vast amount of newly available data opens the way for a broad analysis of different structural characteristics. Additionally, nowadays, the ease and accessibility of predicting protein structures draw many non-experts into the field of structural biology. This makes having accessible tools that help assess the model quality a pressing need. Here, we present AlphaKnot 2.0 which answers that need by giving users an easy-to-use tool for topological analysis of protein structure, and in particular its ability to form knots.

Knots, as protein motifs, were discovered almost 50 years ago (5,6). The motifs were found to be conserved within protein families (7,8), with a single exception being ATC/OTCase family which has both knotted and unknotted proteins (9). For many years, the set of knot types, differing in complexity measured in the number of crossings, to be found in proteins was very limited – to just four possibilities: 3_1_, 4_1_, 5_2_ and 6_1_. Only recently a new type was discovered and verified (3_1_#3_1_) that showed that double knots can exist in natural proteins (10,11). The speed of discovering topologically novel proteins now increased significantly with the power of machine learning algorithms. Analyses based on AlphaFold predictions show that even more types of knotted proteins exist than already known (12,13). There are reports of not only new families but also of new, and often more complex, knot types never before seen in a protein. An excellent example is provided by the protein with 7_1_ knot (UniProtKB ID: Q9PR55; also present in our AlphaKnot database), first predicted by the AlphaFold model (12) and soon confirmed using X-ray diffraction (14). Moreover, these analyses also discovered families with probable dual topology (with both knotted and unknotted members) (15).

The topology of the protein is an important factor in any structure analysis. Studies show that non-trivial topology, such as knots, influence the structure’s ability to withstand degradation (11,16–18) and increase its thermodynamic stability (19–22). Moreover, the knot can be a vital part of the protein’s active site, and provide the cleft for the ligand to bind (23,24). As such, deepening our understanding of how topologically different proteins perform the same function can lead to the conception of novel antimicrobial compounds. One such well-studied pair is a knotted bacterial (TrmD protein) and unknotted eukaryotic (Trm5) methyltransferases. Most of the studies focus on TrmD – a vital enzyme, that is universal for bacteria (also for the ones the World Health Organisation lists as pathogens of global priority due to their antibiotic resistance). There were already several attempts to design inhibitors of the TrmD protein that act by targeting the knot and blocking protein function (25–27). A successful inhibitor that is selective for TrmD (not binding to Trm5) might be the start of a new type of antibiotics. However, even though the knotted proteins are studied from many different perspectives, the fundamental questions remain open, like is there a common role that a knot plays in protein structure? Or what is the evolutionary origin of a knotted structure? Did it emerge from the unknotted protein? We believe that immediate access to protein structures and their topology will aid in answering these important questions.

AlphaKnot 2.0 is an easy-to-use web server that simplifies the analysis of topological characteristics of structure models (proteins, nucleic acids, or other polymers) and gives them visual aids to help understand their complexities. It also includes a database of already analyzed AlphaFold-predicted structures that appear to contain topological knots. The AlphaKnot 2.0 service is free and open to all users and there is no login requirement.

## Materials and methods

### Topology detection

One of the crucial functionalities of the server side of AlphaKnot 2.0 is topology detection. It is realized mainly using the Topoly (28) package, in particular, the HOMFLY-PT polynomial. The user can select the type (and—if applicable—the number) of chain closures (required to transform an open protein chain into a proper mathematical knot) to optimize either computation time, or the accuracy of the result. Similarly, if the user chooses to calculate the knot map (knot matrix), density can also be controlled by the accuracy parameter. As a result we are reporting either the knot type (including composite knots) or ‘Unknown’ for topologies with >12 crossings (appearing in extremely complex structures).

To give more detailed description of the topology, we are also using the knot_pull (29) package to calculate the Dowker-Thistlewaithe (30) notation to describe how does the protein chain, an inherently open curve, realize the closed, mathematical knot. This approach is deterministic, thus there are no additional parameters to specify. The knot_pull tool can be accessed via the submission server for new structures or after using the recompute option for records already available in the database. The results appear in the bottom part of the summary site as a new visualization. The trajectory generated by knot_pull can be easily downloaded using the designated button.

The input file should be in a PDB or mmCIF file format (can be either a single or multi-chain). Alternatively, the user can specify a protein ID from the MGnify database (31), and the corresponding prediction file from the ESM Atlas will be automatically pulled. If the input file contains the pLDDT data in the b-factor column (e.g. mmCIF files from the AlphaFold Model), it will be used for the assessment of the structure, and topological prediction validity. The topology is calculated based on the chain simplified to just the Cα atoms.

### Database of pre-calculated structures

Along with the server, we include a database of pre-analyzed models. It includes all AlphaFold DB v4-predicted protein structures for which models with average full-chain pLDDT value higher or equal to 70 were proposed, and for which non-trivial topology has been detected (>680k structures). This was done in two steps, by first detecting the knot through 100 random chain closures for all high-quality models and then recalculating with 500 closures the structures that appeared to have knots. Additionally, for all knotted (based on AlphaFold predicted structures) structures of <400 amino acids in length, we ran the ESMFold model (2) to generate predicted 3D structures and then applied the same knot identification method as in the case of the AlphaFold structures and compared the resulting topology. Since calculating the full knot maps for all those structures would be infeasible, we have left the users the opportunity to request a more detailed analysis of a database structure through a two-click ‘Recompute in Web Server’ tool. To compare, the first version of the AlphaKnot database had knot maps calculated for all its proteins but it contained topological data of only 21 proteomes from the AlphaFold Database v1.

### Visualization

A customized and extended version of the PDBe Mol* Viewer v3.1 (32) is used for visualizing the protein structures and simplification trajectory from knot_pull. Knot maps (knot matrices) (33) are drawn using Matplotlib (34) library for Python. To help users see the topological complexities in the structure, we are using knot_pull (29) to generate a trajectory of structure simplification steps.

## Server description

### Submission server

**Figure 1.**
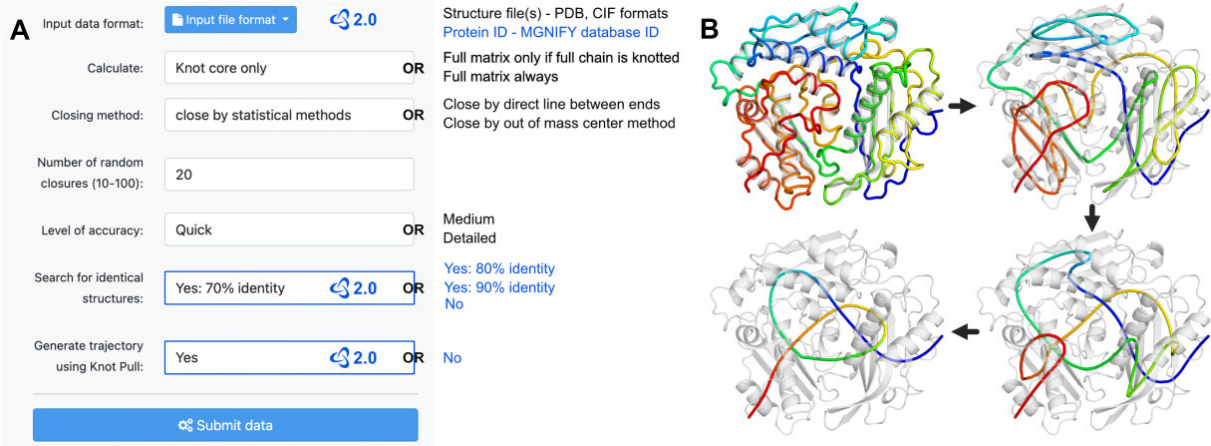
Web Server features. (**A**) Submission options—default and additional parameters. The user can choose from several options of how the topology will be calculated in the structure (including the accuracy). New features provided with the updated AlphaKnot 2.0 are marked with blue and the AlphaKnot’s logo. Please note, that the calculation time is directly related to the selected parameters but also the length and complexity of the structure. For a single structure, it varies from a few seconds to several hours. (**B**) Frames showing simplification of the structure using knot_pull algorithm (based on UniProtKB ID: F1QYU7).

The server performs an analysis of the topology of the input structure which can be given as a PDB/CIF with one or more chains, or a MGnify (31) protein ID. All correctly formatted structure files can be submitted, however, the ones generated with the AlphaFold (or similar) algorithm will return more information, such as quality analysis (based on pLDDT values). The submitted structure will be processed according to the parameters chosen by the user (Figure 1).

The output is a detailed, interactive web page with many different structure visualizations. We present the user with a knot map that shows where (based on the sequence indices) the knots are located within the structure. This matrix shows the protein’s knot fingerprint and is useful for finding slipknots (7,35). The protein chain has a slipknot when it is overall unknotted but has an internal knot (found on any given sub-chain). Many slipknotted proteins are found within transmembrane ion transporters (with S3_1_ topology) (36). Given that the main knot detection algorithm we use is probabilistic, the user can see how the probability cutoff changes the knot landscape on the map using a slider. It is particularly useful for detection of knots of lower probability. Next, the user can mark the position of the knot (knot core) directly on the structure. By using the *pLDDT* button the marked region is colored by pLDDT values to show the quality of the modeling. By using the *Rainbow* button the knotted region is colored in a rainbow gradient (from red to blue). All the knots found by the algorithm within the structure can be visualized this way.

More often than not, the knot is not an easily identifiable part of the protein structure. In some cases, the knot spans hundreds of amino acids, which can be difficult to recognize, even for a trained eye. Therefore, in the updated version of the web server, we use a new tool for simplifying the structure—knot_pull (29). With its trajectory of continuously simpler representation of the structure, one can determine exactly how the knot manifests in the structure. Thanks to the knot type calculations provided by knot_pull, we also give the user more detailed information on where is the gap in the knot (i.e. the implicitly connected ends of the protein chain) based on the crossing order in the Dowker-Thistlewaithe notation. This can facilitate an even more precise comparison between different realizations of the same knot type.

Thanks to the database that exists alongside the server, we now also provide additional information regarding other calculated protein structures. We list the most similar proteins in the AlphaKnot database (by default with 70% sequence identity) and their model’s knot type, enabling a comparison of the topology between the model of the queried protein and its homologs. The topology is thought to be conserved between similar proteins (7,36), thus any differences might indicate that the model is not topologically correct and should be treated cautiously. All new features provided by the web server are easily accessible for all database proteins via a two-click “Recompute in Web Server“ submission button on each protein’s page.

### Database

**Figure 2.**
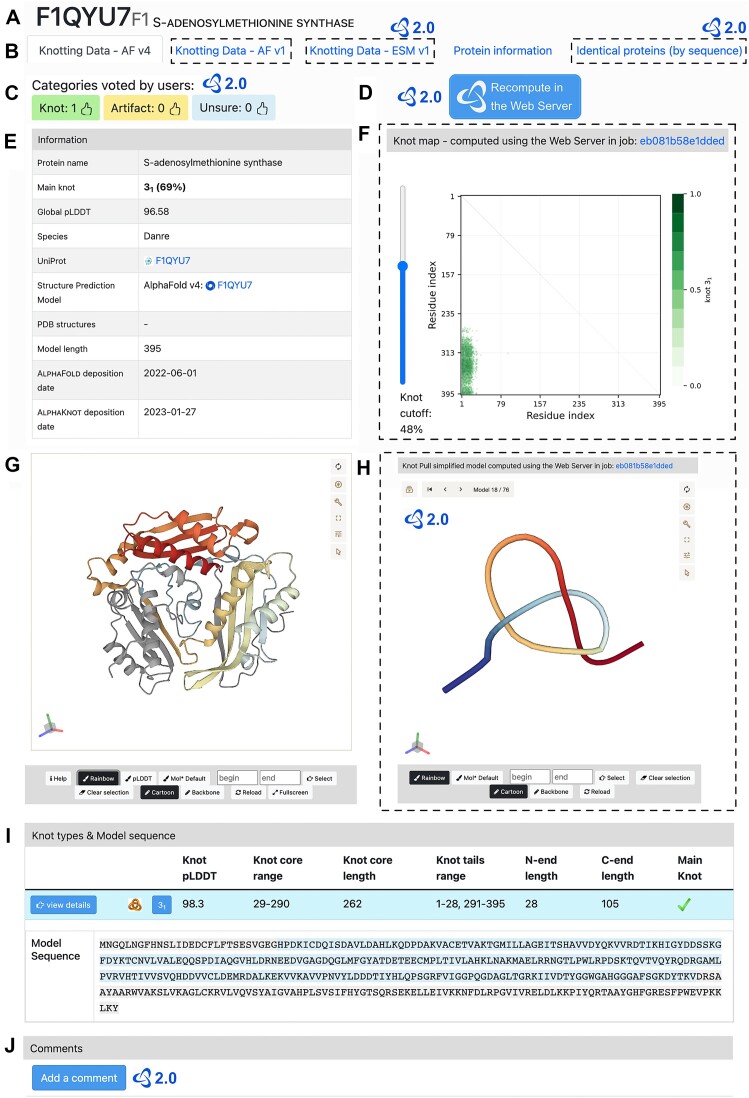
Database protein page—default and additional features. Additional features available after recomputing the model in the Web Server are marked with dashed lines. New features provided with the updated AlphaKnot are marked with the AlphaKnot’s 2.0 logo. (**A**) UniProtKB ID of the protein, model number, and protein name. (**B**) Available tabs. (**C**) Categories voted by users. (**D**) Button for submitting the current model to our Web Server. (**E**) Basic information about the protein and the model. (**F**) knot map with the slider applying different knot probability cutoffs. (**G**) 3D structure of the model. (**H**) Trajectory of simplification steps. (**I**) Information about found knots. (**J**) Comment section.

The size of the database of AlphaKnot (33) increased a hundredfold (from 6000 to >680 000 protein entries). It was done by calculating the topology of most of the models available in the latest (4th) version of the AlphaFold Database (4). To provide high-quality data we processed only models with the average full-chain pLDDT above 70. A minimal protein page consists of two tabs: one with the latest AlphaFold model (*Knotting Data – AF v4*) and the *Protein information* tab. Additional tabs include models of the protein either available in the first version of the AlphaFold Database (*AF v1*) or modeled by us with the ESMFold algorithm (*ESM v1*). Figure 2 shows the contents of a protein page and *Knotting Data – AF v4* tab, with additional elements marked with dashed lines. These will be available after recomputing the protein in the Web Server. From each page, the user can get the most important information about the knot present in the protein model, such as the position of the knot in the structure (*knot core*), its quality (*knot pLDDT*) and type (*main knot*).

## Distinctive features of updated AlphaKnot

The AlphaKnot was updated to include tools that help the user assess the topological correctness of their modeled structure and gather information about all the knotted models predicted with the AlphaFold. In particular, the two components of the AlphaKnot – web server and database, are intrinsically connected. The web server uses the information from the database and the database pages provide a quick job submission to the web server option (to unlock more information about the protein model other than the default, like the knot map, simplified structure, or the topology of homologs).

### Visualization of the simplified structure

Knots in proteins are usually difficult to observe in the 3D structure due to their complexity. In AlphaKnot 2.0 we utilize a tool that gradually simplifies the structure—knot_pull (29). It smooths the protein chain by approximating the process of pulling atoms towards the chain ends (N- and C-terminus). Starting from the native structure, the visualization shows each step of the simplification and ends with a string-like protein chain with a knot. The user can view the visualization in the form of a movie which can be downloaded in the mp4 format, or select a specific frame for closer analysis. Additionally, the structure can be colored with a rainbow gradient for an even easier knot identification.

### Analysis of topology of homologous proteins

AlphaKnot 2.0 analyzes knotted homologous proteins of the queried protein submitted in the Web Server and returns their knot types. In particular, this feature can be used to find knotted homologs to new proteins with *de novo* designed sequences. The homology is based on sequence identity (user-specified parameter with the default value of 70%). The analysis is done locally using the MMseqs2 tool (37) and is based on proteins available in AlphaKnot’s database, thus knotted and with an average model pLDDT >70. As a result, the user is provided with a list with direct links to the homologs in the database, and information about their topology (AlphaFold model and ESMFold model where available) and length. This feature allows an easy way of noticing differences in knot types of similar proteins, which might be an indication of incorrect modeling. This is especially important in the field of knotted proteins since the machine learning algorithms were trained on a small number of such proteins and might produce artifacts leading to non-trivial topologies (13).

### Information about >680 000 knotted AlphaFold models

The database of AlphaKnot is now expanded to contain hundreds of thousands of proteins, which models predicted by AlphaFold are knotted. Due to a significant number of new entries, manual verification of the models (which we did for the AlphaKnot 1.0) is no longer possible. However, we believe that any manual confirmation of the automatically generated models will benefit the user. Therefore, with the new version, we enabled users to leave comments on each protein model and to vote by choosing one of the three categories *Knot*, *Unsure* and *Artifact*.

### Extensive database filtering

The vast number of knotted models in the AlphaKnot 2.0 database provides an opportunity for large-scale analyses of knotted proteins. For convenient access to the data we store, the user can specify precisely what they seek by using the expanded *Advanced search* functionality, which applies filters on the database entries and is also available as an API. In particular, there are three main categories for the queries, that relate to the information about the protein, the model, or the knot. The search criteria include protein name, gene name, taxonomy (kingdom, family, organism), and cross-references (to UniProt, InterPro, Pfam, PDB and EC databases) for the protein. For model-related searches, the user can specify average pLDDT values (either for the whole chain or only the knotted region), model category, and chain length. Lastly, the criteria for the knot are its type, probability, and knot core’s range and length. It is possible to join the search criteria with logical operators (AND, OR NOT).

### Comparison to other services

As for the server section of the AlphaKnot 2.0 Database, we are aware of an alternative: ‘Protein Knot server’ which also allows users to detect topology in the provided protein structure (38). The service provided via AlphaKnot 2.0, however, provides the user with much more flexibility in parameters, allowing for the detection of not only knots (with two different methods) but also slipknots and the calculation of the full knot map and simplified structure via Knot_pull. Additional tools may also help with differentiating artifacts and identification of similar proteins already available in the database. For data already available in AlphaKnot 2.0, the user is also provided with additional biological annotations. The ‘KnotGenome’ (39) on the other hand allows for topological detection in chromosomal data and ‘KymoKnot’ (40) for linear and circular polymers. We are also aware of the ‘PyKnot’ plugin for visualization and characterization of knots in proteins (41). Also in this case the user cannot detect slipknots and construct knot maps, limiting analysis capabilities. As for the alternatives for the Topoly package (28), we acknowledge the respective list of the following packages: ‘GISA’ (42), ‘Knoto-ID’ (43), ‘SKMT algorithm’ (44) and ‘TEPPP’ (45).

From the database perspective, the closest available alternative is the KnotProt Database (46). In comparison, however, the AlphaKnot Database analyses all proteins provided in the AlphaFold Database (protein predictions) instead of the PDB Database (protein structures obtained from the experimental methods) thus differing both in data type and size. Additionally, while both servers provide the option to calculate the topology of the given protein structure, AlphaKnot boasts more options to choose from including more detailed parameters and the use of the simplification algorithm. From further alternatives, we acknowledge ‘PconsFam’ which also provides simplified topological information for Pfam families’ representatives (47). In this database, protein structure predictions are coming from the CONFOLD algorithm (48) and the focus of the database is shifted more towards predicting contact maps.

## Summary

AlphaKnot 2.0 is a web server for detailed visualization of knotting in user-provided structures, as well as a database of entanglements in AlphaFold-predicted protein models. This updated service offers several new features that aid with the analysis of the topology and help the user assess the topological correctness of their query.

The web server gives detailed topological information on the structure (provided by the user as either a mmCIF- or PDB-formatted file), as well as a visual guide to understand it with a trajectory of simplification steps calculated by the knot_pull package. Additionally, it is integrated with the AlphaKnot database to provide information about knot types of proteins (from the database) sequentially similar to the query. Given that topology should be conserved between similar proteins, if the query has a different knot type it might indicate that the model is not topologically correct and should be treated with caution. All new features provided by the web server are easily accessible for all database proteins via a two-click job submission button on each protein page.

The database of AlphaKnot was also significantly updated. By using data from the latest (4th) version of the AlphaFold Database, we expanded the number of knotted models to more than 680 000 (1 140 000 models including those generated with ESMFold). With such a vast amount of data, manual verification is not possible, thus we provide the users with the option to leave a comment and vote (choosing ‘Knot’, ‘Artifact’ or ‘Unsure’) on each model. To further help the user, each protein shorter than 400 amino acids has an ESMFold model generated to allow for comparison between predictions of these two ML methods.

## Data Availability

AlphaKnot 2.0 is available at https://alphaknot.cent.uw.edu.pl/. It is free and open to all users without login requirement.

## References

[1] Jumper J., Evans R., Pritzel A., Green T., Figurnov M., Ronneberger O., Tunyasuvunakool K., Bates R., Žídek A., Potapenko A. et al. Highly accurate protein structure prediction with AlphaFold. Nature. 2021; 596:583–589. 34265844 10.1038/s41586-021-03819-2 PMC8371605

[2] Lin Z., Akin H., Rao R., Hie B., Zhu Z., Lu W., Smetanin N., Verkuil R., Kabeli O., Shmueli Y. et al. Evolutionary-scale prediction of atomic-level protein structure with a language model. Science. 2023; 379:1123–1130. 36927031 10.1126/science.ade2574

[3] Baek M., DiMaio F., Anishchenko I., Dauparas J., Ovchinnikov S., Lee G.R., Wang J., Cong Q., Kinch L.N., Schaeffer R.D. et al. Accurate prediction of protein structures and interactions using a three-track neural network. Science. 2021; 373:871–876. 34282049 10.1126/science.abj8754 PMC7612213

[4] Varadi M., Anyango S., Deshpande M., Nair S., Natassia C., Yordanova G., Yuan D., Stroe O., Wood G., Laydon A. et al. AlphaFold Protein Structure Database: massively expanding the structural coverage of protein-sequence space with high-accuracy models. Nucleic Acids Res. 2022; 50:D439–D444. 34791371 10.1093/nar/gkab1061 PMC8728224

[5] Sulkowska J.I. On folding of entangled proteins: knots, lassos, links and -curves. Curr. Opin. Struc. Biol. 2020; 60:131–141. 10.1016/j.sbi.2020.01.007 32062143

[6] Hsu S.T.D. Folding and functions of knotted proteins. Curr. Opin. Struc. Biol. 2023; 83:102709. 10.1016/j.sbi.2023.102709 37778185

[7] Sulkowska J.I., Rawdon E.J., Millet K.C., Onuchic J. N., Stasiak A. Conservation of complex knotting and slipknotting patterns in proteins. Biophys. J. 2012; 102:E1715–E1723. 10.1073/pnas.1205918109 PMC3387036 22685208

[8] Zayats V., Sikora M., Perlinska A.P., Stasiulewicz A., Gren B.A., Sulkowska J.I. Conservation of knotted and slipknotted topology in transmembrane transporters. Biophys. J. 2023; 122:4528–4541. 37919904 10.1016/j.bpj.2023.10.031 PMC10719070

[9] Virnau P., Mirny L.A., Kardar M. Intricate knots in proteins: function and evolution. PLoS Comput. Biol. 2006; 2:e122. 16978047 10.1371/journal.pcbi.0020122 PMC1570178

[10] Perlinska A.P., Nguyen M.L., Pilla S., Staszor E., Lewandowska I., Bernat A., Purta E., Augustyniak R., Bujnicki J.M., Sulkowska J.I. Are there double knots in proteins? Prediction and *in vitro* verification based on TrmD-Tm1570 fusion from *C. nitroreducens*. Front. Mol. Biosci. 2023; 10:1223830. 38903539 10.3389/fmolb.2023.1223830 PMC11187310

[11] Bruno da Silva F., Lewandowska I., Kluza A., Niewieczerzal S., Augustyniak R., Sulkowska J. I. First crystal structure of double knotted protein TrmD-Tm1570–inside from degradation perspective. 2023; bioRxiv doi:14 March 2023, preprint: not peer reviewed 10.1101/2023.03.13.532328.

[12] Brems M.A., Runkel R., Yeates T.O., Virnau P. AlphaFold predicts the most complex protein knot and composite protein knots. Protein Sci. 2022; 31:e4380. 35900026 10.1002/pro.4380 PMC9278004

[13] Perlinska A.P., Niemyska W.H., Gren B.A., Bukowicki M., Nowakowski S., Rubach P., Sulkowska J.I. AlphaFold predicts novel human proteins with knots. Protein Sci. 2023; 32:e4631. 36960558 10.1002/pro.4631 PMC10108431

[14] Hsu M.F., Sriramoju M.K., Lai C.H., Chen Y.R., Huang J.S., Ko T.P., Huang K.F., Hsu S.T.D. Structure, dynamics, and stability of the smallest and most complex 7_1_ protein knot. J. Biol. Chem. 2024; 300:105553. 38072060 10.1016/j.jbc.2023.105553 PMC10840475

[15] Sikora M., Klimentova E., Uchal D., Sramkova D., Perlinska A.P., Nguyen M.L., Korpacz M., Malinowska R., Nowakowski S., Rubach P. et al. Knot or Not? Identifying unknotted proteins inknotted families with sequence-based ML model. 2024; bioRxiv doi:07 September 2023, preprint: not peer reviewed 10.1101/2023.09.06.556468. PMC11184937 38888487

[16] San Martín Á., Rodriguez-Aliaga P., Molina J.A., Martin A., Bustamante C., Baez M. Knots can impair protein degradation by ATP-dependent proteases. Proc. Natl. Acad. Sci. U.S.A. 2017; 114:9864–9869. 28847957 10.1073/pnas.1705916114 PMC5604015

[17] Fonseka H., Javidi A., Oliveira L., Micheletti C., Stan G. Unfolding and translocation of knotted proteins by clp biological nanomachines: Synergistic contribution of primary sequence and topology revealed by molecular dynamics simulations. J. Phys. Chem. B. 2021; 125:7335–7350. 34110163 10.1021/acs.jpcb.1c00898

[18] Wang H., Li H. Mechanically tightening, untying and retying a protein trefoil knot by single-molecule force spectroscopy. Chem. Sci. 2020; 11:12512–12521. 34123232 10.1039/d0sc02796k PMC8162576

[19] Sriramoju M.K., Yang T.J., Hsu S.T.D. Comparative folding analyses of unknotted versus trefoil-knotted ornithine transcarbamylases suggest stabilizing effects of protein knots. Biochem. Biophys. Res. Commun. 2018; 503:822–829. 29920242 10.1016/j.bbrc.2018.06.082

[20] Wojciechowski M., Gómez-Sicilia À., Carrión-Vázquez M., Cieplak M. Unfolding knots by proteasome-like systems: simulations of the behaviour of folded and neurotoxic proteins. Mol. BioSyst. 2016; 12:2700–2712. 27425826 10.1039/c6mb00214e

[21] Sułkowska J., Sułkowski P., Szymczak P., Cieplak M. Stabilizing effect of knots on proteins. Proc. Natl. Acad. Sci. U.S.A. 2008; 105:19714–19719. 19064918 10.1073/pnas.0805468105 PMC2604914

[22] Rivera M., Hao Y., Maillard R., Baez M. Mechanical unfolding of a knotted protein unveils the kinetic and thermodynamic consequences of threading a polypeptide chain. Sci. Rep. 2020; 10:9562. 32533020 10.1038/s41598-020-66258-5 PMC7292828

[23] Christian T., Sakaguchi R., Perlinska A.P., Lahoud G., Ito T., Taylor E.A., Yokoyama S., Sulkowska J.I., Hou Y.M. Methyl transfer by substrate signaling from a knotted protein fold. Nat. Struct. Mol. Biol. 2016; 23:941–948. 27571175 10.1038/nsmb.3282 PMC5429141

[24] Dabrowski-Tumanski P., Stasiak A., Sulkowska J. In search of functional advantages of knots in proteins. PLoS One. 2016; 11:e0165986. 27806097 10.1371/journal.pone.0165986 PMC5091781

[25] Zhong W., Koay A., Ngo A., Li Y., Nah Q., Wong Y.H., Chionh Y.H., Ng H.Q., Koh-Stenta X., Poulsen A. et al. Targeting the bacterial epitranscriptome for antibiotic development: discovery of novel tRNA-(N1G37) methyltransferase (TrmD) inhibitors. ACS Infect. Dis. 2019; 5:326–335. 30682246 10.1021/acsinfecdis.8b00275

[26] Hill P.J., Abibi A., Albert R., Andrews B., Gagnon M.M., Gao N., Grebe T., Hajec L.I. J., Huang L.I., Livchak S. et al. Selective inhibitors of bacterial t-RNA-(N1G37) methyltransferase (TrmD) that demonstrate novel ordering of the lid domain. J. Med. Chem. 2013; 56:7278–7288. 23981144 10.1021/jm400718n

[27] Whitehouse A.J., Thomas S.E., Brown K.P., Fanourakis A., Chan D.S.H., Libardo M.D.J., Mendes V., Boshoff H.I.M., Floto R.A., Abell C. et al. Development of inhibitors against Mycobacterium abscessus tRNA (m1G37) methyltransferase (TrmD) using fragment-based approaches. J. Med. Chem. 2019; 62:7210–7232. 31282680 10.1021/acs.jmedchem.9b00809 PMC6691401

[28] Dabrowski-Tumanski P., Rubach P., Niemyska W., Gren B., Sulkowska J. Topoly: Python package to analyze topology of polymers. Brief. Bioinform. 2020; 22:bbaa196. 10.1093/bib/bbaa196 PMC8138882 32935829

[29] Jarmolinska A.I., Gambin A., Sulkowska J.I. Knot_pull—python package for biopolymer smoothing and knot detection. Bioinformatics. 2020; 36:953–955. 31504154 10.1093/bioinformatics/btz644 PMC9883683

[30] Dowker C.H., Thistlethwaite M.B. Classification of knot projections. Topol. Appl. 1983; 16:19–31.

[31] Richardson L., Allen B., Baldi G., Beracochea M., Bileschi M., Burdett T., Burgin J., Caballero-Pérez J., Cochrane G., Colwell L. et al. The microbiome sequence data analysis resource in 2023. Nucleic Acids Res. 2022; 51:D753–D759. 10.1093/nar/gkac1080 PMC9825492 36477304

[32] Sehnal D., Bittrich S., Deshpande M., Svobodová R., Berka K., Bazgier V., Velankar S., Burley S., Koča J., Rose A. Mol* Viewer: modern web app for 3D visualization and analysis of large biomolecular structures. Nucleic Acids Res. 2021; 49:W431–W437. 33956157 10.1093/nar/gkab314 PMC8262734

[33] Niemyska W., Rubach P., Gren B., Nguyen M., Garstka W., Silva F., Rawdon E., Sulkowska J. AlphaKnot: server to analyze entanglement in structures predicted by AlphaFold methods. Nucleic Acids Res. 2022; 50:W44–W50. 35609987 10.1093/nar/gkac388 PMC9252816

[34] Hunter J. Matplotlib: A 2D graphics environment. Comput. Sci. Eng. 2007; 9:90–95.

[35] King N.P., Yeates E.O., Yeates T.O. Identification of rare slipknots in proteins and their implications for stability and folding. J. Mol. Biol. 2007; 373:153–166. 17764691 10.1016/j.jmb.2007.07.042

[36] Zayats V., Sikora M., Perlinska A.P., Stasiulewicz A., Gren B.A., Sulkowska J.I. Conservation of knotted and slipknotted topology in transmembrane transporters. Biophys. J. 2023; 122:4528–4541. 37919904 10.1016/j.bpj.2023.10.031 PMC10719070

[37] Steinegger M., Söding J. MMseqs2 enables sensitive protein sequence searching for the analysis of massive data sets. Nat. Biotechnol. 2017; 35:1026–1028. 29035372 10.1038/nbt.3988

[38] Kolesov G., Virnau P., Kardar M., Mirny L. Protein knot server: detection of knots in protein structures. Nucleic Acids Res. 2007; 35:W425–W428. 17517776 10.1093/nar/gkm312 PMC1933242

[39] Sulkowska J., Niewieczerzal S., Jarmolinska A., Siebert J., Virnau P., Niemyska W. KnotGenome: a server to analyze entanglements of chromosomes. Nucleic Acids Res. 2018; 46:W17–W24. 29905836 10.1093/nar/gky511 PMC6030981

[40] Tubiana L., Polles G., Orlandini E., Micheletti C. KymoKnot: a web server and software package to identify and locate knots in trajectories of linear or circular polymers. Eur. Phys. J. E. Soft Matt. 2018; 41:72. 10.1140/epje/i2018-11681-0 29884956

[41] Lua R. PyKnot: a PyMOL tool for the discovery and analysis of knots in proteins. Bioinformatics. 2012; 28:2069–2071. 22611132 10.1093/bioinformatics/bts299

[42] Grønbæk C., Hamelryck T., Røgen P. GISA: using Gauss Integrals to identify rare conformations in protein structures. PeerJ. 2020; 8:e9159. 32566389 10.7717/peerj.9159 PMC7293858

[43] Dorier J., Goundaroulis D., Benedetti F., Stasiak A. Knoto-ID: a tool to study the entanglement of open protein chains using the concept of knotoids. Bioinformatics. 2018; 34:3402–3404. 29722808 10.1093/bioinformatics/bty365

[44] Bale A., Rambo R., Prior C. The SKMT algorithm: a method for assessing and comparing underlying protein entanglement. PLoS Comput. Biol. 2023; 19:e1011248. 38011290 10.1371/journal.pcbi.1011248 PMC10703313

[45] Herschberg T., Pifer K., Panagiotou E. A computational package for measuring Topological Entanglement in Polymers, Proteins and Periodic systems (TEPPP). Comput. Phys. Commun. 2023; 286:108639.

[46] Dabrowski-Tumanski P., Rubach P., Goundaroulis D., Dorier J., Sułkowski P., Millett K., Rawdon E., Stasiak A., Sulkowska J. KnotProt 2.0: a database of proteins with knots and other entangled structures. Nucleic Acids Res. 2018; 47:D367–D375. 10.1093/nar/gky1140 PMC6323932 30508159

[47] Lamb J., Jarmolinska A., Michel M., Menéndez-Hurtado D., Sulkowska J., Elofsson A. PconsFam: an interactive database of structure predictions of pfam families. J. Mol. Biol. 2019; 431:2442–2448. 30796988 10.1016/j.jmb.2019.01.047

[48] Adhikari B., Bhattacharya D., Cao R., Cheng J. CONFOLD: residue-residue contact-guidedab initioprotein folding. Proteins: Struct. Funct. Bioinform. 2015; 83:1436–1449. 10.1002/prot.24829 PMC4509844 25974172

